# Hungarian and Indonesian rice husk as bioadsorbents for binary biosorption of cationic dyes from aqueous solutions: A factorial design analysis

**DOI:** 10.1016/j.heliyon.2023.e17154

**Published:** 2023-06-09

**Authors:** Hadid Sukmana, Gergő Ballai, Tamás Gyulavári, Erzsébet Illés, Gábor Kozma, Zoltán Kónya, Cecilia Hodúr

**Affiliations:** a Doctoral School of Environmental Science, University of Szeged, Moszkvai krt. 9, Szeged 6725, Hungary; b Department of Applied and Environmental Chemistry, University of Szeged, Rerrich Béla tér. 1, Szeged 6720, Hungary; c Department of Food Engineering, University of Szeged, Mars tér. 7, Szeged 6724, Hungary; d Department of Biosystems Engineering, University of Szeged, Moszkvai krt. 9, Szeged 6725, Hungary

**Keywords:** Binary biosorption, Bioadsorbents, Dyes, Factorial design, Rice husk

## Abstract

The wastewater of the dye industry can be characterized by a complex chemical composition and consists of numerous dyes. Bioadsorbents are increasingly applied for the biosorption of dyes because they are inexpensive and environmentally friendly. Rice husk (RH) is a potential agricultural waste that can be converted into a bioadsorbents for the biosorption of cationic dyes. Herein, the removal of methylene blue (MB) and basic red 9 (BR9) dyes by Hungarian rice husk (HRH) and Indonesian rice husk (IRH) using binary biosorption was investigated. Adsorbents were characterized by zeta potential, Fourier-transform infrared spectroscopy, and scanning electron microscopy. Batch biosorption evaluated the influence of different variables, including pH, adsorbent dose, contact time, and initial concentrations. Several factors that influence the biosorption of MB and BR9 onto rice husk were assessed using main effect, Pareto charts, normal probability plots, and interaction effect in a factorial design. The optimum contact time was 60 min. Isotherm and kinetic models of MB and BR9 in binary biosorption fitted to the Brunauer–Emmett–Teller multilayer and the Elovich equation based on correlation coefficients and nonlinear chi-square. Results showed that the biosorption capacity of HRH was 10.4 mg/g for MB and 10 mg/g for BR9; values for IRH were 9.3 mg/g and 9.6 mg/g, respectively. Therefore, HRH and IRH were found to be effective adsorbents for removing MB and BR9 via binary biosorption.

## Graphical abstract

**Figure:**
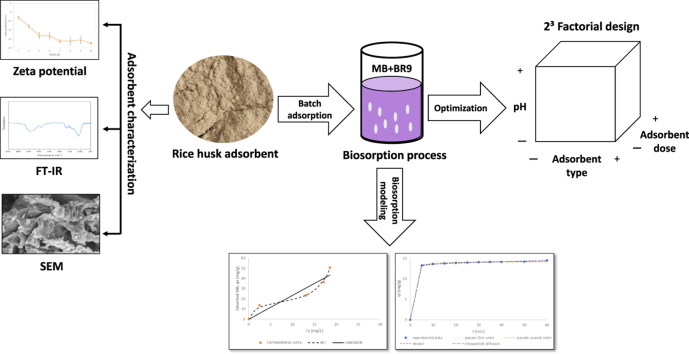

## 1 Introduction

The dye industry continues to develop and expand in many countries. However, dyes are chemically stable and poorly degradable owing to their complex structures [1]. Textile, leather, and printing industries discharge dye-containing wastewater into the environment, causing ecological problems [2]. Dye-containing wastewater produced by the textile industry is dangerous and toxic [3]. Dye contamination is associated with various diseases in animals and humans [4].

Cationic dyes such as methylene blue (MB) and basic red 9 (BR9), are commonly used in the textile industry. MB can be used in cotton, paper, silk, and wool dyeing. However, long-term exposure to MB can have negative effects such as diarrhea, nausea, anemia, vomiting, and hypertension [5,6]. BR9 is utilized to color nylon, silk, wool, and acrylic. This dye has poor biodegradation and carcinogenic effects when directly released into the natural environment [7,8].

Wastewater treatment techniques, such as liquid membrane filtration, coagulation-flocculation, oxidation, biodegradation, and biosorption, are used to remove dye [9]. Biosorption is an effective, low-cost alternative with simple operational conditions and relatively low energy demand [10].

Agricultural waste is widely investigated for wastewater treatment, and rice husk is a promising candidate in this regard. This waste can be explored as a low-priced, efficient, and eco-friendly adsorbent. Indonesia is one of the world’s largest rice producers. However, not all dry grains become rice during production. In 2022, 11.134 million tons (as estimated) of rice husk were produced [11]. However, the application of rice husk is still limited, and it predominantly becomes waste. Besides, Hungarian rice husks are used for composting, brick, and animal feed. Reusing rice husks as bioadsorbents can reduce the generation of waste in the agricultural sector.

Most studies on dye biosorption by bioadsorbents have concentrated on single-component biosorption. Studying binary biosorption is more complex, as well as more parameters are needed to describe the process [12]. Textile industrial effluent contains several dyes that complicate biosorption due to various interactions and competition between adsorbates and adsorbents. Studies on the binary biosorption of dyes using multiple bioadsorbents, such as cellulose-based cotton fiber and activated carbon from kiwi, cucumber, and potato peels, have been reported [[13], [14], [15]]. Based on these, it can be expected that the suitability of bioadsorbents will be extensively investigated in subsequent binary biosorption studies.

To our knowledge, this is the first report on the removal of MB and BR9 in binary dye solutions using different origins of raw rice husks. Further, in the present work, we investigated the feasibility of using Hungarian rice husk (HRH) and Indonesian rice husk (IRH) as bioadsorbents for the binary biosorption of MB and BR9 cationic dyes from aqueous solutions. Rice husk can adsorb dyes efficiently provided by the biosorption capabilities of its carboxylic and phenolic groups [16]. Moreover, it is insoluble in water and exhibits a granular structure, useful mechanical properties, and high chemical stability, making it suitable for wastewater treatment [17]. We characterized the functional group of RH, surface morphology, and surface charge of RH using Fourier-transform infrared spectroscopy (FT-IR), scanning electron microscopy (SEM), and zeta potential analysis. Biosorption isotherm and kinetic models were applied to characterize the binary biosorption of MB and BR9. The factorial design analysis was used to optimizing pH, dose, and type of adsorbent to examine the efficiency and interactions during binary biosorption.

## 2 Materials and methods

### 2.1 Dyes

**Fig. 1:**
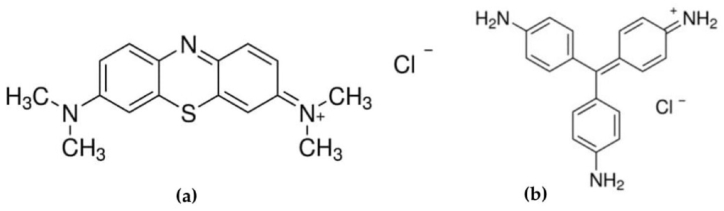
Structures of (a) methylene blue and (b) basic red 9.

MB (Molar Chemical) and BR9 (Sigma-Aldrich) were diluted using stock solutions (1000 mg/L) and mixed. The structures of MB and BR9 are shown in Fig. 1(a) and (b). The pH of dye solutions was adjusted using sodium hydroxide (NaOH) and hydrochloric acid (HCL) solutions. The chemical reagents with analytical grades were used in this study.

### 2.2 Adsorbent preparation

Rice husk (RH; raw material) was collected from Nagykun 2000 Mezőgazdasági Zrt., Hungary, and Cianjur City, Indonesia. RH was washed three to four times with distilled water and dried in an oven at 105 °C for 2 h. RH was crushed and sieved for a particle size fraction of <250 μm.

### 2.3 Characterization of adsorbent

#### 2.3.1 Zeta potential analysis

A Nano ZS (Malvern, UK) dynamic light scattering apparatus with a 4 mW He−Ne laser source (λ = 633 nm) was used for zeta potential measurements. RH suspensions were placed in disposable zeta cells (DTS1070) and analyzed at 25 °C. Suspensions were prepared as follows: 10 mg of RH was added into 10 mL of sodium chloride (0.01 M) at various pH. The pHs were adjusted before measurement with 0.1 M HCl or 0.1 M NaOH and checked after the analysis.

#### 2.3.2 FT-IR and SEM analysis

Fourier-transform infrared spectroscopy (FT-IR) measurements were carried out with a Bruker Vertex 70 instrument (16 scans/s, 4 cm^−1^ resolution) using the KBr pellet technique. The morphology of the adsorbent surface was investigated with a Hitachi S-4700 Type II scanning electron microscope (SEM) using an accelerating voltage of 10 kV.

### 2.4 Batch biosorption

Batch biosorption experiments were conducted to assess the effect of pH (within the 3–7 pH range), adsorbent dose (250, 375, and 500 mg), contact time (5–60 min), and initial concentration (between 30 and 120 mg/L) for MB and BR9 removal in binary biosorption. Further, 250 mL of binary dye solutions with a constant 25 °C temperature and stirring speed of 100 rpm were applied for the experiment. Biosorption isotherms were investigated using different initial concentrations of each dye between 30 and 120 mg/L for 500 mg of RH, at pH 7 and 25 °C. Samples were taken every 5 min to evaluate biosorption kinetics. Important factors that influenced MB and BR9 biosorption were identified using 23 factorial designs in Minitab® 20 statistical software (Table 1). Initial concentrations of binary dye solutions (30 mg/L for each dye), stirring speed (100 rpm), and optimum time (60 min) were kept constant.

**Table 1:** Factorial designs for binary dye solutions.

Factor	Coded symbol	Low level (−1)	High level (+1)
Adsorbent type	A	IRH	HRH
pH	B	3	7
Dose	C	250	500

The binary dye solutions and RH adsorbent were separated by centrifugation for 15 min at 4000 rpm. UV–vis spectrophotometer (Biochrom WPA Lightwave II) was used to analyze solutions. The absorbance of MB and BR9 in binary dye solutions were measured at 664 and 545 nm, respectively. Binary dye removal (%) was calculated as equation (1):

(1) $$Removal=\frac{c_{i}-c_{e}}{c_{i}}100$$

where *c*_*i*_ (mg/L) is the initial dye concentration, and *c*_e_ (mg/L) is the final dye concentration. The adsorbed amounts of binary dye solutions were calculated as equation (2):

(2) $$q_{e}=\left(c_{i}-c_{e}\right)\;\frac{V}{m}$$

where *q*_*e*_ is the adsorbate amount on the adsorbent; *c*_*i*_ (mg/L) is the initial concentrations of dyes in binary biosorption; *c*_*e*_ (mg/L) is the equilibrium concentrations of dyes in binary biosorption; *V* (L) is the volume of binary dye solutions; and *m* (g) is mass of rice husk adsorbent. The experimental data was applied to biosorption isotherm and kinetic models and was chosen based on the best correlation coefficient (R^2^) and nonlinear chi-square (χ^2^).

## 3 Results and discussion

### 3.1 pH-dependent surface charge

**Fig. 2:**
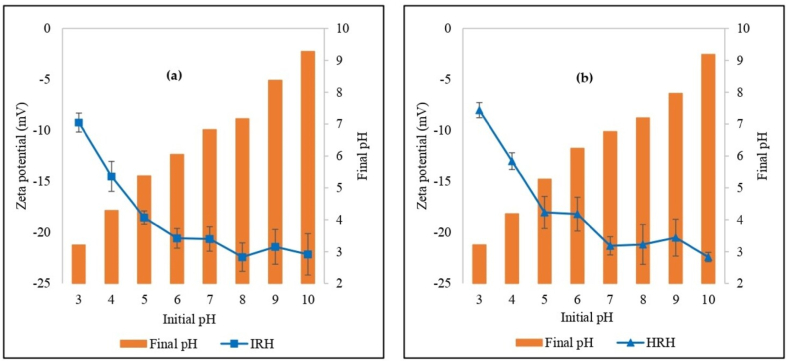
pH dependence of zeta potential for (a) Indonesian rice husk and (b) Hungarian rice husk.

Zeta potential measurements provide information on electrokinetic potential and are used to characterize surface charge and predict nanoparticle stability in a colloid dispersion [18] under various conditions (pH, electrolyte concentration). Zeta potential changes depending on pH and ionic strength [19] (Fig. 2(a) and (b)). Zeta potentials were negative at all pH values. The negative charge on the surface increased with the increased pH (i.e., basic pH 3–10). This condition was generated by the deprotonation of functional groups on the RH surfaces. [20,21]. Increasing the negative charge is expected to increase the removal of cationic dyes due to electrostatic interaction [22].

### 3.2 FT-IR and SEM analysis

**Fig. 3:**
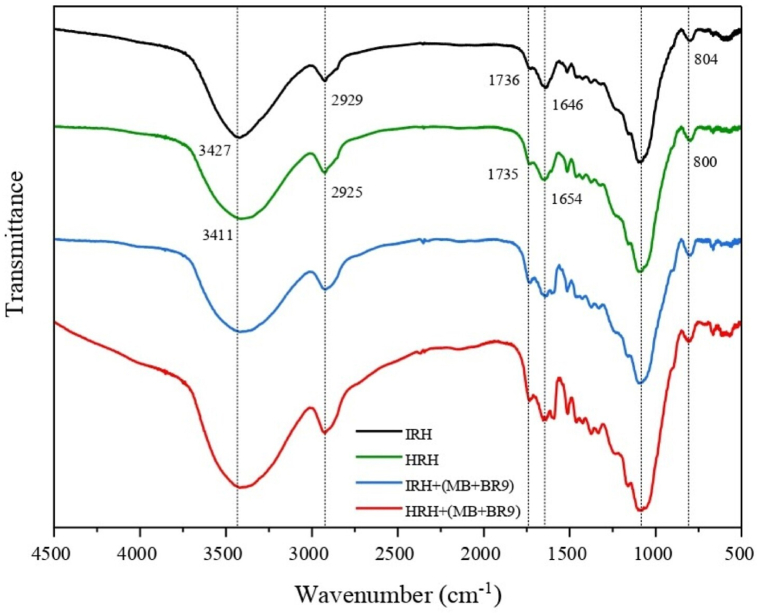
Fourier-transform infrared spectroscopy absorption spectra band values for the Hungarian rice husk and Indonesian rice husk before and after biosorption.

FT-IR analysis were conducted to characterize the RH samples before and after the biosorption of dyes (Table 2 and Fig. 3). The bands observed were similar to the ones investigated by Antil et al. [23]. Before biosorption, the band at 3427 cm^−1^ (IRH) and 3411 cm^−1^ (HRH) is due to O–H bonds stretching in lignocellulose materials. The bands at 2929 cm^−1^ (IRH) and 2925 cm^−1^ (HRH) can be attributed to C–H bonds stretching. Further, the bands at 1736 cm^−1^ (IRH) and 1735 cm^−1^ (HRH) due to C=O bonds stretching of aldehyde groups in the hemicellulose component [24]. The bands at 1646 cm^−1^ (IRH) and 1654 cm^−1^ (HRH) can be attributed to O–H bonds. The bands at 1102 cm^−1^ (IRH) and 1099 cm^−1^ (HRH) can be ascribed to stretching Si–O–Si bonds. Finally, the bands at 804 cm^−1^ (IRH) and 800 cm^−1^ (HRH) refer to the presence of Si–O bonds in the rice husk structure. After the biosorption of MB and BR9, the original band positions shifted, and new bands appeared. The band at 1602 cm^−1^ due to C=N and C=O bonds stretches. The bands at 1334 cm^−1^ and 1166 cm^−1^ can be attributed to C–N bonds stretching: the former band is specific for dimethylamino groups [25].

**Table 2:** FT-IR absorption bands of RH.

Absorption band (cm^−1^)				
IRH	HRH	IRH (MB + BR9)	HRH (MB + BR9)	Assignment
3427	3411	3413	3422	O–H and N–H
2929	2925	2927	2927	C–H, –CH_3_ or –CH_2_
1736	1735	1733	1738	C=O
1646	1654	1647	1652	O–H
–	–	1602	1602	C=N and C=O
–	–	1334	1334	C–N
–	–	1166	1166	C–N
1102	1099	1098	1098	Si–O–Si
804	800	803	808	Si–O

**Fig. 4:**
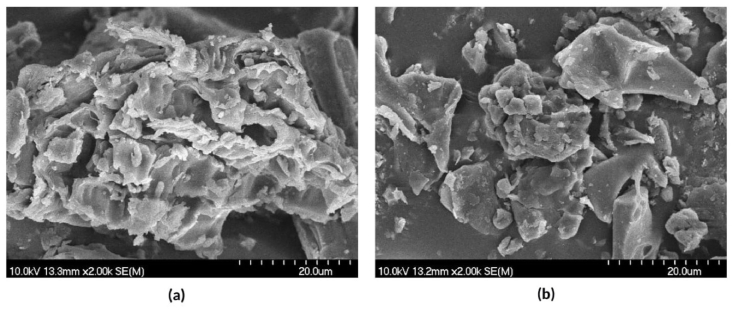
Scanning electron microscopy images of (a) HRH, and (b) IRH.

SEM analysis were used to examine the surface morphology of RH (Fig. 4(a) and (b)). Surfaces were highly irregular and could not be characterized by any well-defined morphology. Rough surfaces can be attributed to silica dispersed in the bulk, which is common for this material [26,27].

### 3.3 Effect of pH

**Fig. 5:**
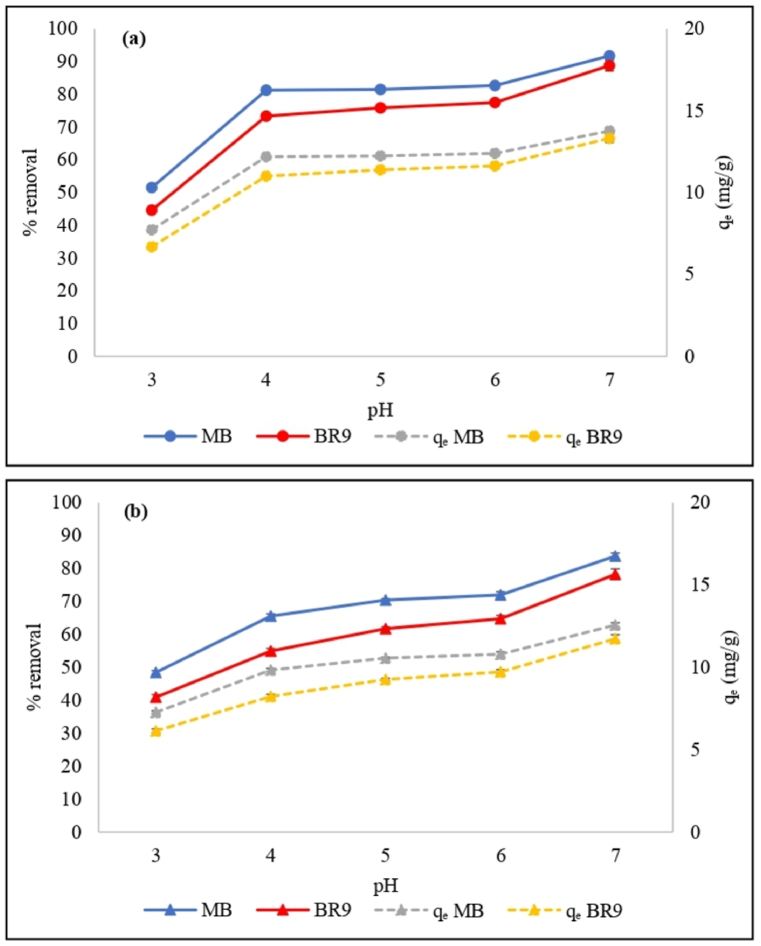
Effect of pH on methylene blue and basic red 9 removals by HRH (a) and IRH (b) using the following parameters: 500 mg of RH, 30 mg/L initial concentration, 60 min biosorption time, and 25 °C temperature. (For interpretation of the references to color in this figure legend, the reader is referred to the Web version of this article.)

The effect of pH was studied to determine the optimal pH for removing MB and BR9. The removal percentages of these dyes by HRH and IRH increase with the increase of pH from 3 to 7 (Fig. 5(a) and (b)). Generally, removal percentages for biosorption of cationic dyes will increase at high pH values (basic condition) and decrease at low pH values (acidic condition) [28]. At lower pH values the removal percentages are lower due to more H^+^ ions in the solution. The H^+^ ions compete for the adsorbent sites with cationic dyes during the biosorption process [29]. Meanwhile, under basic conditions, the adsorbent is more negatively charged. Hence, the electrostatic interaction between the positively charged of cationic dye molecules and the negatively charged of RH adsorbent increases the removal percentage [30].

### 3.4 Effect of adsorbent dose

**Fig. 6:**
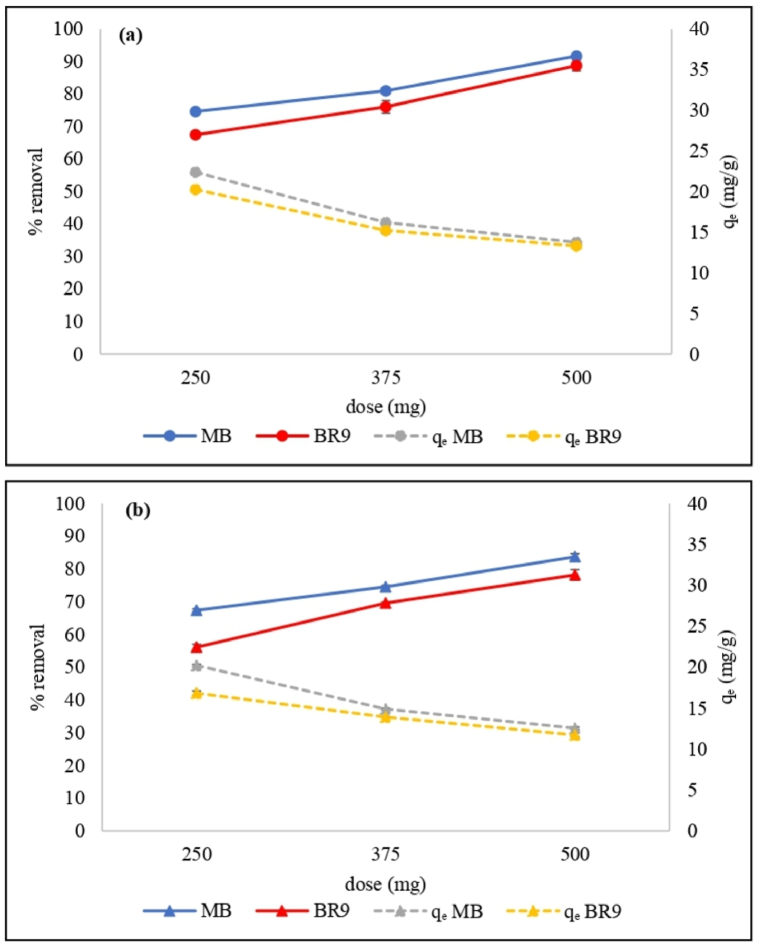
Effect of adsorbent dose on methylene blue and basic red 9 removals by HRH (a) and IRH (b) using the following parameters: 30 mg/L initial concentration, pH 7, 60 min biosorption time, and 25 °C temperature. (For interpretation of the references to color in this figure legend, the reader is referred to the Web version of this article.)

The effect of the adsorbent dose was also examined (Fig. 6(a) and (b)). At the highest adsorbent dose (500 mg), MB and BR9 removal percentages by HRH were as high as 91.7% and 88.8%, respectively. For IRH, these values were 83.8% and 78.2%, respectively. However, the biosorption capacities for MB and BR9 by HRH decreased from 22.4 to 13.8 mg/g and from 20.3 to 13.3 mg/g, respectively. Meanwhile, using IRH, the biosorption capacities were decreased from 20.2 to 12.6 mg/g for MB and from 16.8 to 11.7 mg/g for BR9. The increasing RH dose increases the removal percentage and decreases the biosorption capacities due to more available unoccupied adsorbent sites on the RH surface [31]. Based on these results, 500 mg of RH was selected as the optimal adsorbent dose and used in a subsequent experiments.

### 3.5 Effect of contact time

**Fig. 7:**
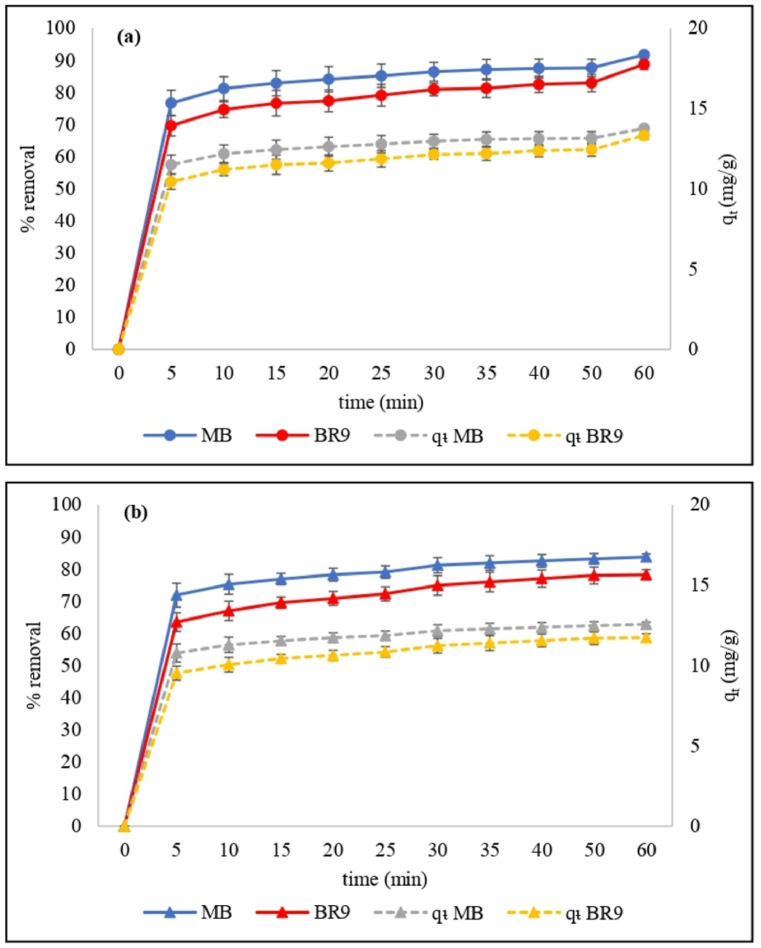
Effect of contact time on methylene blue and basic red 9 removal by HRH (a) and IRH (b) using the following parameters: 500 mg of RH, pH 7, 30 mg/L initial concentration, 25 °C temperature. (For interpretation of the references to color in this figure legend, the reader is referred to the Web version of this article.)

Biosorption varies with the contact time, and contact time is vital to dye removal efficiency (Fig. 7(a) and (b)). Increasing the contact time from 0 to 60 min increases the MB and BR9 removal from binary dyes solution due to increased interaction probability of MB and BR9 dyes with the surface of RH bioadsorbent [32]. Removal of MB and BR9 on HRH or IRH was initially rapid because of the more available biosorption sites [33]. Removal within 5 min using HRH was 76.7% for MB and 69.7% for BR9. In the same timeframe, removal by IRH was 71.9% for MB and 63.5% for BR9. After the initial removal, as active sites were increasingly occupied biosorption gradually slowed and stabilized [34]. Biosorption equilibrium was reached within 60 min. Moreover, removal rates by HRH were found to be 91.7% for MB and 88.8% for BR9 and 83.8% for MB, and 78.2% for BR9 by IRH.

### 3.6 Effect of initial concentration

**Fig. 8:**
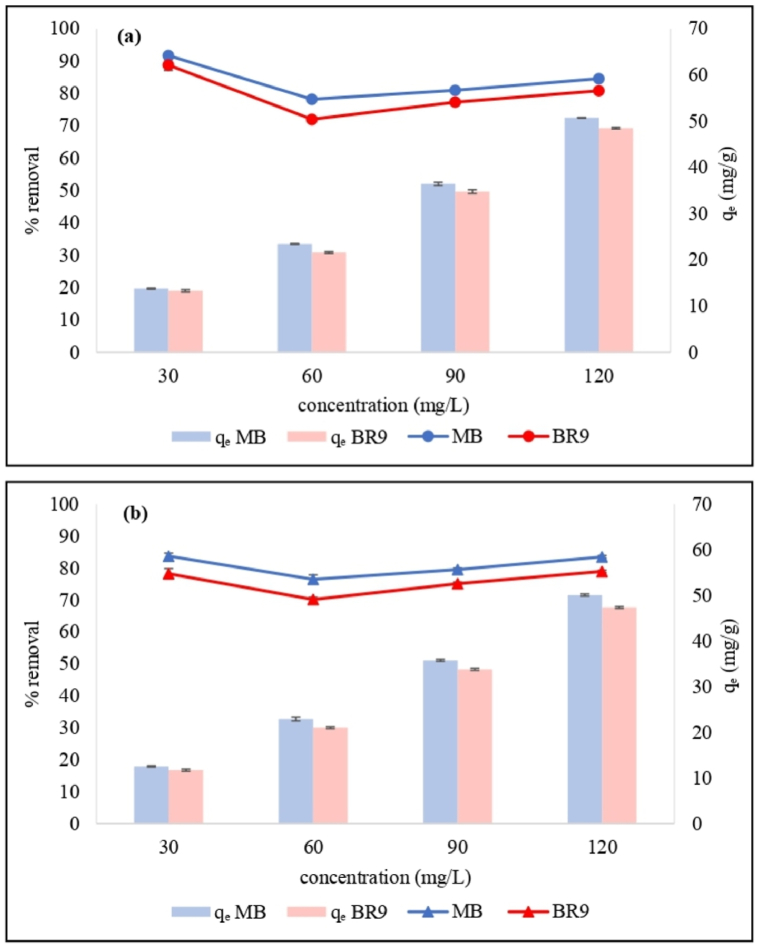
Effect of initial dye concentration on methylene blue and basic red 9 removals by HRH (a) and IRH (b) using the following parameters: 500 mg of RH, pH 7, 60 min biosorption time, and 25 °C temperature. (For interpretation of the references to color in this figure legend, the reader is referred to the Web version of this article.)

The impact of various initial concentrations of dye (30, 60, 90, and 120 mg/L) was assessed to explore biosorption capacity (Fig. 8(a) and (b)). Biosorption at equilibrium increased with increasing initial concentration of dye. The biosorption capacities of HRH for 120 mg/L MB and BR9 were 50.7 and 48.5 mg/g, respectively, and 50.1 and 47.4 mg/g for IRH, respectively. At lower initial dye concentrations, fewer dye molecules were adsorbed, as expected [35]. Increasing MB and BR9 concentration led to lower active sites of the bioadsorbent owing to the high biosorption rate of MB and BR9 at the beginning of the biosorption process [36]. Additionally, increasing the initial dye concentrations increased mass transfer during biosorption; therefore, higher equilibrium values were obtained, thereby reducing the removal percentage of dyes [36,37].

### 3.7 Modeling of biosorption isotherm and kinetics

Biosorption isotherm and kinetic of MB and BR9 were constructed to identify the binary biosorption mechanisms. Langmuir and Brunauer–Emmett–Teller (BET) multilayer isotherm models were evaluated using varying initial dye concentrations, amounts of RH, and temperature.

**Fig. 9:**
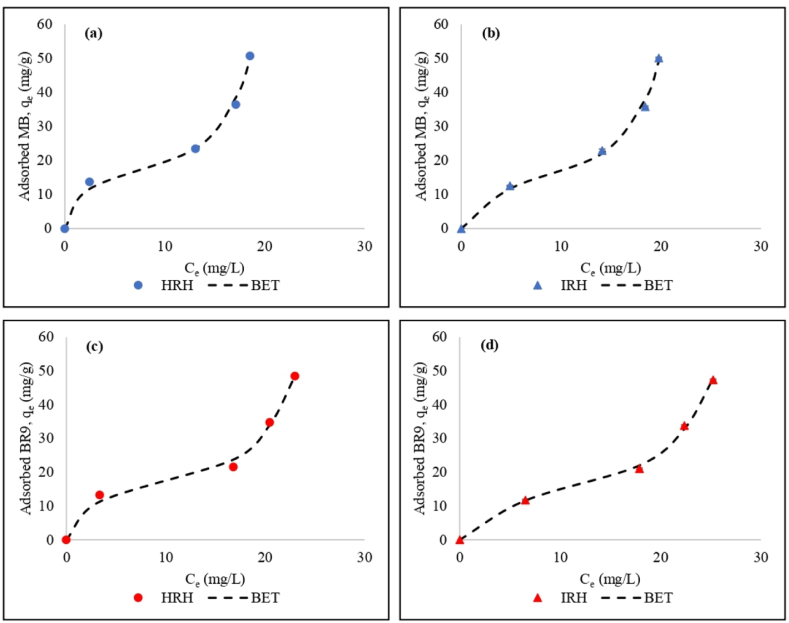
Comparison of experimental data and BET multilayer isotherm models for (a) MB using HRH, (b) MB using IRH, (c) BR9 using HRH, and (d) BR9 using IRH.

The type II isotherms (S-shaped) were obtained in all cases, and the BET multilayer model could be fitted to the experimental data (Fig. 9(a)–(d)). The point of saturation was not reached because the dyes were first adsorbed as a monolayer; then, a multilayer was formed [38]. The BET multilayer model is one of the most common equations applied for type II isotherms. In contrast, the Langmuir model is mostly used for type I isotherms, which refers to monolayer biosorption [39].

On the other hand, ionic dyes tend to undergo self-association (aggregation) in aqueous solutions. Ionic dyes can form dimers, trimers, and micelles during biosorption. Several factors can influence this condition, including pH, concentration, temperature, etc. [40]. In a previous research, MB formed aggregates below 3.4 × 10^−5^ M [41], while BR9 was liable to form aggregations and dimers between concentrations of 10^−2^ and 10^−5^ M [42]. The result show that MB and BR9 tend to form dimers and aggregates in aqueous solution at concentrations of 6.2 × 10^−5^ M (∼20 mg/L) (Fig. 9(a) and (b)) and 8.7 × 10^−5^ M (∼25 mg/L) (Fig. 9(c) and (d)), respectively.

The Langmuir isotherm describes the biosorption process at homogeneous adsorbent sites [43]. The Langmuir model equation [44] is calculated as equation (3):

(3) $$q_{e}=\frac{Q_{m}K_{L}C_{e}}{1+K_{L}C_{e}}$$

where *C*_*e*_ is the concentration at equilibrium, *q*_*e*_ is the adsorbate amount on the adsorbent, *Q*_*m*_ is the amount required for monolayer biosorption, and *K*_*L*_ is the Langmuir isotherm constant related to the energy of biosorption. The extended Langmuir model [45] was used to evaluate binary biosorption as follows:

(4) $$q_{ei}=\frac{{{K_{Li}Q}_{mi}C}_{ei}}{1+\sum K_{Li}C_{ei}}$$

Based on equation (4), the individual equilibrium capacity for the binary biosorption of MB and BR9 is calculated as equations (5), (6):

(5) $$q_{e1}=\frac{{{K_{L1}Q}_{m1}C}_{e1}}{1+K_{L1}C_{e1}+K_{L2}C_{e2}}$$

(6) $$q_{e2}=\frac{{{K_{L2}Q}_{m2}C}_{e2}}{1+K_{L1}C_{e1}+K_{L2}C_{e2}}$$

where *q*_*e1*_ and *q*_*e2*_ are the amounts of solutes (MB and BR9) adsorbed by the adsorbent (mg/g); *C*_*e1*_ and *C*_*e2*_ are adsorbate concentrations at equilibrium (mg/L); *Q*_*m1*_ and *Q*_*m2*_ represent the maximum biosorption capacities (mg/g); and *K*_*L1*_ and *K*_*L2*_ are the Langmuir constants (L/mg).

The BET isotherm model characterizes multilayer biosorption and is derived from the generalized Langmuir model [39]. The liquid phase biosorption of BET multilayer isotherm equation [46] is given as equation (7):

(7) $$q_{e}=Q_{m}\frac{K_{S}C_{eq}}{\left(1-K_{L}C_{eq}\right)\left(1-K_{L}C_{eq}+K_{S}C_{eq}\right)}$$

where *Q*_*m*_ is the amount required for monolayer biosorption, *K*_*S*_ is the biosorption constant for the first layer, and *K*_*L*_ is the biosorption constant for the upper layers.

The BET multilayer isotherm model best fit experimental results based on correlation coefficients (R^2^) and nonlinear chi-square (χ^2^) (Table 3). This model shows that the biosorption of MB and BR9 formed more than one layer of adsorbate on RH surfaces [47]. The difference in the Q_m_ value in Table 3 is because the Langmuir isotherm model supposes the biosorption process occurred only on a monolayer. Therefore, the Q_m_ calculated in the BET model is less than the Q_m_ calculated in the Langmuir model [48]. The Langmuir model uses the same value for monolayer and maximum biosorption capacities. However, the monolayer and maximum biosorption capacities are different for the BET multilayer model. The maximum biosorption capacity for the BET multilayer model can be infinite [46]. This can be proven by comparing the Q_m_ values between the Langmuir and BET multilayer isotherm. Based on these results, the Q_m_ calculated by the BET multilayer model agrees with the q_e_ experimental after reaching equilibrium, which were 12.6–13.8 mg/g for MB and 11.7–13.3 mg/g for BR9 (Table 4).

**Table 3:** Nonlinear isotherm parameters for MB and BR9 during binary biosorption.

Model	Parameter	Indonesian Rice Husk		Hungarian Rice Husk	
		Methylene blue	Basic Red 9	Methylene blue	Basic Red 9
Extended Langmuir	Q_m_ (mg/g) (10^5^)	9.8	9.6	6.1	9.9
	K_L_ (10^−6^)	2.2	1.7	3.9	1.8
	R^2^	0.834	0.844	0.764	0.744
	χ^2^	1.07	0.97	1.52	1.59
BET	Q_m_ (mg/g)	9.3	9.6	10.4	10
	K_L_	0.04	0.03	0.04	0.03
	K_S_	1.3E+04	2.5	2E+06	1.5E+07
	R^2^	0.991	0.997	0.987	0.987
	χ^2^	0.06	0.02	0.08	0.08

**Table 4:** Nonlinear kinetic parameters for MB and BR9 during binary biosorption.

Model	Parameter	Indonesian Rice Husk		Hungarian Rice Husk	
		Methylene blue	Basic Red 9	Methylene blue	Basic Red 9
Experimental	q_e_ (mg/g)	12.6	11.7	13.8	13.3
Pseudo-first-order	q_e_ (mg/g)	12.1	11.1	12.9	12.1
	k_1_	0.4	0.3	0.4	0.4
	R^2^	0.545	0.844	0.574	0.788
	χ^2^ (10^−2^)	1.2	2.1	1.1	2.3
Pseudo-second-order	q_e_ (mg/g)	12.5	11.8	13.4	12.8
	k_2_ (10^−2^)	8.4	6	8	6.1
	R^2^	0.883	0.955	0.868	0.915
	χ^2^ (10^−3^)	3	6.2	3.6	9.1
Elovich equation	α	2.6E+07	1.4E+05	2.7E+07	4.3E+05
	Β	1.7	1.4	1.6	1.4
	R^2^	0.931	0.967	0.913	0.943
	χ^2^ (10^−3^)	1.7	4.6	2.3	6.2

K_S_ was higher than K_L_, which showed that the affinity of the first layer between the RH surface and dyes is greater than in the second layer [49]. Similar results were obtained by the BET model for the removal of dyes using different bioadsorbents, such as sugarcane bagasse [40], soybean hull [50], and banana pseudostem [51].

Table 5 compares the biosorption capacities of HRH and IRH for cationic dyes in binary dye solutions from various adsorbents. The result shows that the raw material of HRH and IRH provides good biosorption capacities. Utilization of HRH and IRH without chemical/physical modification can reduce energy and chemical consumption during bioadsorbent preparation. In addition, rice husk as a low-cost bioadsorbent is abundantly available in large quantities, has eco-friendly components, and is excellent in the regeneration–reusability for removing dyes [52]. Therefore, HRH and IRH become promising alternative bioadsorbent compared to other adsorbent materials and have the potential for cationic dyes removal from binary dye solutions.

**Table 5:** Comparison of the biosorption capacity for cationic dyes in binary dye solutions using various adsorbents.

No	Adsorbent	Adsorbate	Biosorption capacity (mg/g)	Reference
1	Medlar nut activated carbon	Basic yellow 28	37	[53]
		Methylene blue	135.2	
2	Ziziphus mauritiana nut activated carbon	Basic blue 41	190.8	[54]
		Basic yellow 28	133.1	
3	Cellulose-based modified citrus peels	Methylene blue	795.1	[33]
		Crystal violet	884.1	
4	Cotton–graphene oxide composite	Methylene blue	35.7	[55]
		Crystal violet	19.2	
5	Functionalized microcrystalline cellulose	Methylene blue	100.2	[14]
		Neutral red	76.7	
6	Bombax buonopozense bark	Basic blue 41	75.1	[56]
		Safranin	80.6	
7	Hungarian rice husk	Methylene blue	10.4	**This study**
		Basic red 9	10	
8	Indonesian rice husk	Methylene blue	9.3	**This study**
		Basic red 9	9.6	

To describe the binary biosorption mechanism, we used three kinetic models, such as the pseudo-first-order [57], pseudo-second-order kinetics [58], and the Elovich equation [59] (Equations (8)–(10)):

(8) $$q_{t}=q_{e}\;\left(1-e^{{-k}_{1}t}\right)$$

(9) $$q_{t}=\frac{q_{e}^{2}k_{2}t}{1+k_{2}q_{e}t}$$

(10) $$q_{t}=\frac{1}{\beta }\ln\left(1+\alpha \beta t\right)$$

where *q*_*e*_ is the adsorbate amount on the adsorbent (mg/g) at equilibrium; *q*_*t*_ represents the adsorbate amount on the adsorbent (mg/g) at time *t* (min); *k*_*1*_ is the constant of first-order (L/min); *k*_*2*_ is the constant of second-order (g/mg/min); *α* represent the Elovich equation constant (mg/g/min); and *β* (mg/g) is the constant of desorption.

The pseudo-first-order (PFO) and pseudo-second-order (PSO) models were applied to understand the biosorption kinetic behavior. PFO and PSO are commonly used to define biosorption mechanisms in physical biosorption (physisorption) and chemical biosorption (chemisorption) processes, respectively [60]. PFO and PSO models did not result in the best coefficient correlations and nonlinear chi-squares (χ^2^) (Table 4). Besides, the calculated q_e_ value obtained from both models was lower than the experimental q_e_ value.

**Fig. 10:**
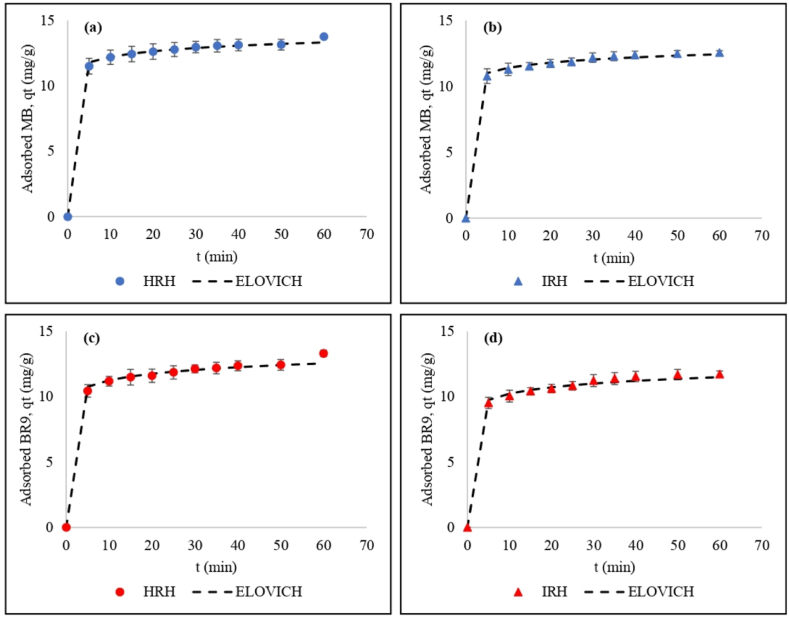
Comparison of experimental data and Elovich equation model for (a) MB using HRH, (b) MB using IRH, (c) BR9 using HRH, and (d) BR9 using IRH.

The Elovich model describes the biosorption process that occurs quickly at the initial stage, which then decreases over time due to the activation energy changes on the adsorbent surface [61]. This equation describes a biosorption mechanism for a heterogeneous adsorbent [10,59,62]. The α value for MB was higher than for BR9, indicating faster initial biosorption [63]. According to correlation coefficients (R^2^) and nonlinear chi-square (χ^2^) for nonlinear kinetic models for binary biosorption indicated the Elovich equation as the best fit (Table 4 and Fig. 10(a)–(d)). Based on this result, chemisorption took place during biosorption. Other researchers also applied the Elovich equation to analyze the biosorption kinetics of MB [61] and BR9 [64].

PFO and PSO models cannot describe the diffusion mechanism on the binary biosorption of MB and BR9. Therefore, the intra-particle model was applied to determine the rate-limiting of dye biosorption onto RH adsorbent. The intra-particle diffusion model [65] is calculated as equation (11):

(11) $$q_{t}=k_{id}\sqrt{t}+C$$

**Fig. 11:**
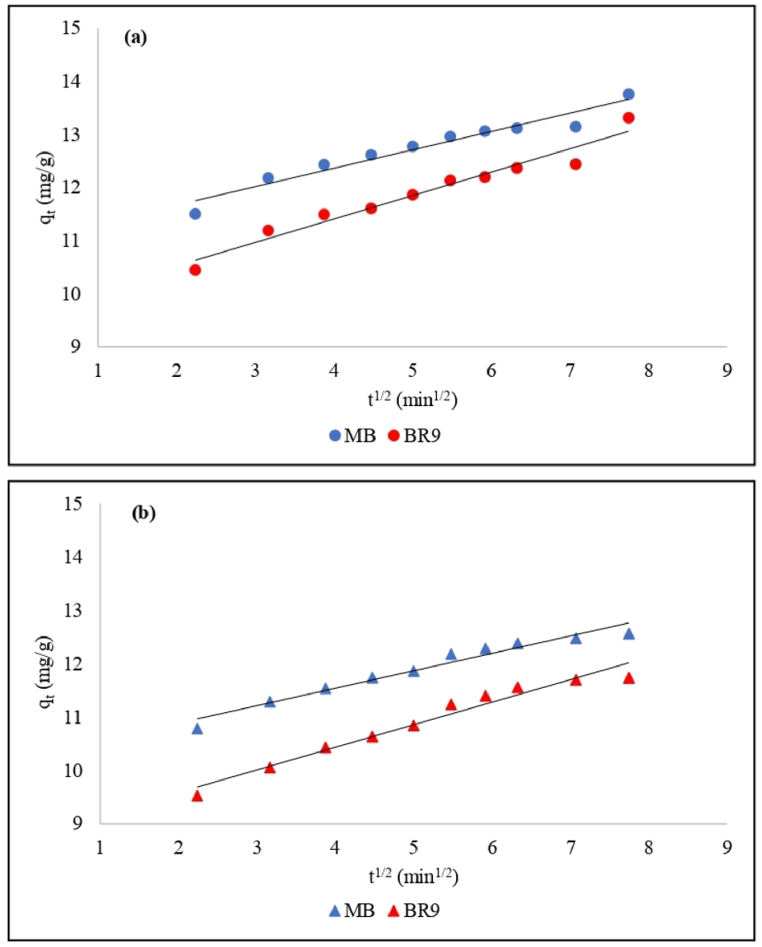
Intra-particle diffusion model for (a) HRH, and (b) IRH.

Here, *k*_*id*_ is the constant of intra-particle diffusion (mg/g/min), and *C* is a constant describing the boundary layer thickness. The linearized intra-particle diffusion kinetic model was applied to plot q_t_ vs t^1/2^; the fitted (straight) line did not fit the experimental (Fig. 11(a) and (b)). This result indicated a rate-limiting step of biosorption, which did not the occur only by intra-particle diffusion [66]. Hence, the biosorption of MB and BR9 occurred in three different steps.

Firstly, the solution transfers MB and BR9 to the surface of the rice husk adsorbent. Secondly, intra-particle diffusion takes place as the dyes reach the pores of the rice husk adsorbent. The strength of biosorption for monolayer and multilayer are different in this stage. Biosorption strength was higher for monolayer biosorption because the adsorbate easily adsorbs on the surface of the rice husk [67]. This condition can be confirmed based on the BET isotherm model, which yielded the higher value for the first layer constant (K_S_) compared to those for the upper layers of adsorbates on the adsorbent (K_L_). The last step is the equilibrium stage [68]. The C value was not equal to zero (C value range of 8.7–11 mg/g in Table 6), which implies that mass transfer and intra-particle diffusion contributed the most during the removal of MB and BR9 in binary biosorption [5,65].

**Table 6:** Intra-particle diffusion parameters for MB and BR9 during binary biosorption.

Model	Parameter	Indonesian Rice Husk		Hungarian Rice Husk	
		Methylene blue	Basic Red 9	Methylene blue	Basic Red 9
Intra-particle diffusion	k_id_	0.3	0.4	0.3	10.4
	C	10.2	8.7	11	9.6

### 3.8 Biosorption mechanism

**Fig. 12:**
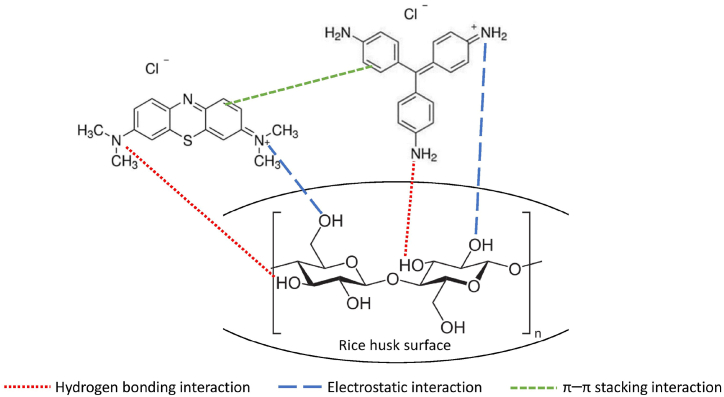
The possible interactions between the rice husk adsorbent (cellulose unit) and dyes in binary biosorption.

The mechanism of biosorption can be explained by considering the structure of the adsorbates and the surface characteristics of the adsorbents. Both MB and BR9 are cationic dyes and dissociate into MB⁺ + Cl⁻ ions and BR9⁺ + Cl⁻ ions in aqueous solutions. Besides, rice husk is a lignocellulose comprising cellulose, hemicellulose, lignin, silica, and other minor components [69]. These components form many functional groups on the rice husk surface, and it becomes effective bioadsorbent due to their availability and low cost for application in wastewater treatment. Cellulose has many polar O and H atoms that play important roles in intramolecular and intermolecular hydrogen bonding [70]. Therefore, the possible interactions between rice husk adsorbents (cellulose unit) and dyes in binary biosorption are hydrogen bonding, electrostatic, and π–π stacking, as shown in Fig. 12.

Hydrogen bonding interactions occur between the surface O–H groups of rice husk and –(N(CH_3_)_2_) groups of dyes [71]. Functional groups such as phenolic and carboxyl groups have a strong negative charge evident from zeta potential analysis and can interact with MB and BR9 by electrostatic interactions [5,66]. Further, the benzene ring in MB and BR9 possibly forms a π–π stacking interaction during binary biosorption. Besides, due to binary biosorption, the competitive biosorption of the dyes can take place. Thus, an adsorbent site on the surface of the rice husk could be partially overlapped with MB and BR9 [14].

### 3.9 Factorial design analysis

Factorial design analysis was used to evaluate factors for MB and BR9 removal in binary biosorption. The factorial design allows significant factors to be retained and insignificant factors to be neglected. This property reduces the number of experiments required. However, applying such an approach results in higher removal percentages [72]. Influences on the biosorption of MB and BR9 onto HRH and IRH were evaluated considering main effects, Pareto charts, normal probability plots, and interaction effects (Table 7).

**Table 7:** Matrix and results of the 2^3^ full factorial design.

Run number	Codes value of variables			Removal (%)		Standard deviation	
	Adsorbent Type	pH	Dose	MB	BR9	MB	BR9
1	−1	−1	−1	47.1	39.4	0.3	0.6
2	1	−1	−1	48.0	40.6	0.3	0.6
3	−1	1	−1	67.4	56.1	0.5	0.9
4	1	1	−1	74.7	67.5	0.2	0.7
5	−1	−1	1	48.5	40.9	0.6	0.8
6	1	−1	1	51.5	44.6	0.3	0.2
7	−1	1	1	83.8	78.2	0.9	1.6
8	1	1	1	91.7	88.8	0.8	1.7

**Fig. 13:**
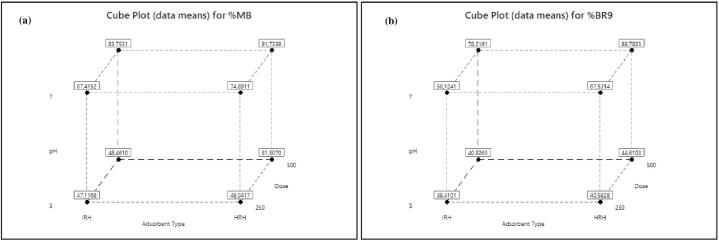
Cube plots of dye removals for (a) MB (%) and (b) BR9 (%) in binary biosorption.

Further, cube plots are provided to illustrate interactions of each factor (Fig. 13(a) and (b)). These plots show that increasing pH from 3 to 7 and adsorbent from 250 to 500 mg enhances removal significantly for both dyes. In addition, HRH was the more efficient absorbent. The highest removal was 91.7% for MB and 88.8% for BR9 at pH 7 using 500 mg of HRH.

The main effect, interaction effect, coefficients, standard deviations, regression coefficients, *T*, and probability (*P*) values are provided in Table 8. The main factors (adsorbent type, pH, dose) for both dyes were significant (*p* < 0.05). At the same time, all two-way interactions for MB and BR9 in binary biosorption were significant, excluding two-way interactions (adsorbent type*dose) for BR9. However, three-way interactions for MB and BR9 were not significant. When a factor effect is positive, removal efficiency increases at high levels; when negative, removal efficiency decreases from low to high levels [73]. Further, the model exhibited an adjusted R^2^ of 99.89% for MB and 99.71% for BR9, which satisfied the statistical model.

**Table 8:** Estimated effects and coefficients for MB and BR9 during binary biosorption.

Term	Effect	Coefficient	SE Coefficient	T-Value	P-Value	VIF
**MB dye**						
Constant		64.1	0.1	560.7	0	
Adsorbent Type	4.8	2.4	0.1	21.0	0	1
pH	30.6	15.3	0.1	133.9	0	1
Dose	9.5	4.8	0.1	41.8	0	1
Adsorbent Type*pH	2.8	1.4	0.1	12.3	0	1
Adsorbent Type*Dose	0.7	0.4	0.1	3.1	0	1
pH*Dose	7.1	3.6	0.1	31.3	0	1
Adsorbent Type*pH*Dose	−0.4	−0.2	0.1	−1.6	0.1	1
S	0.56					
R^2^	99.92%					
Adjusted R^2^	99.89%					
Predicted R^2^	99.83%					
**BR9 dye**						
Constant		57.0	0.2	281.3	0	
Adsorbent Type	6.7	3.4	0.2	16.5	0	1
pH	31.3	15.6	0.2	77.1	0	1
Dose	12.2	6.1	0.2	30.1	0	1
Adsorbent Type*pH	4.3	2.1	0.2	10.6	0	1
Adsorbent Type*Dose	0.4	0.2	0.2	1.0	0.3	1
pH*Dose	9.5	4.7	0.2	23.3	0	1
Adsorbent Type*pH*Dose	−0.8	−0.4	0.2	−2.1	0.1	1
S	0.99					
R^2^	99.80%					
Adjusted R^2^	99.71%					
Predicted R^2^	99.54%					

Dye removal efficiencies (%) are calculated after discarding adsorbent type*pH*dose (A*B*C) interactions for both dyes and neglecting adsorbent type*dose (A*C) interactions for BR9 as follows:

(12) MBremoval=38.28–2.182A+2.297B−0.03324C+0.7052A*B+0.002824A*C+0.014287B*C

(13) BR9removal=35.03–1.995A+0.733B−0.04561C+1.070A*B+0.018900B*C

Equations (12), (13) describe how experimental variables and their interactions influence dye biosorption. Positive values indicate that dye removal increases when the effect increases and vice-versa [74]. Therefore, as adsorbent type (A) increased from low to high, percentage removal decreased to 2.182% for MB and to 1.995% for BR9. Also, as pH (B) increased from low to high, MB and BR9 removal increased to 2.297% and 0.733%, respectively. Finally, as adsorbent dose (C) was increased from low to high, percentages decreased by 0.03324% for MB and by 0.04561% for BR9.

Interaction effects can be evaluated by the analysis of variance (ANOVA). ANOVA separates variation into its components (Table 9). ANOVA provides the sum of squares to evaluate the contributions of different factors. F ratios compare respective mean–square–effects to mean–square–error, and *p* values provide the lowest significance level that leads to the null hypothesis refusal [75]. Main factors (adsorbent type, pH, dose) and two-way interactions were highly significant (*p* < 0.05) and corroborated with the model and experimental results at a 95% confidence level [76]. Conversely, three-way interactions were insignificant (*p* > 0.05).

**Table 9:** Analysis of variance for MB and BR9 during binary biosorption.

Source	DF	Adjusted SS	Adjusted MS	F-Value	P-Value
**MB dye**					
Model	7	6667.7	952.5	3037.9	0
Linear	3	6310.1	2103.4	6708.3	0
Adsorbent Type	1	138.6	138.6	442.1	0
pH	1	5624.5	5624.5	17938.3	0
Dose	1	547.0	547.0	1744.5	0
2-Way Interactions	3	356.9	119.0	379.4	0
Adsorbent Type*pH	1	47.8	47.8	152.3	0
Adsorbent Type*Dose	1	3.0	3.0	9.5	0.01
pH*Dose	1	306.2	306.2	976.4	0
3-Way Interactions	1	0.8	0.8	2.4	0.1
Adsorbent Type*pH*Dose	1	0.8	0.8	2.4	0.1
Error	16	5.0	0.3		
Total	23	6672.8			
**BR9 dye**					
Model	7	7688.1	1098.3	1113.3	0
Linear	3	7037.2	2345.7	2377.7	0
Adsorbent Type	1	270.0	270.0	273.6	0
pH	1	5871.0	5871.0	5951.1	0
Dose	1	896.2	896.2	908.4	0
2-Way Interactions	3	646.7	215.6	218.5	0
Adsorbent Type*pH	1	109.9	109.9	111.4	0
Adsorbent Type*Dose	1	1.1	1.1	1.1	0.3
pH*Dose	1	535.8	535.8	543.1	0
3-Way Interactions	1	4.2	4.2	4.3	0.1
Adsorbent Type*pH*Dose	1	4.2	4.2	4.3	0.1
Error	16	15.8	1.0		
Total	23	7703.9			

**Fig. 14:**
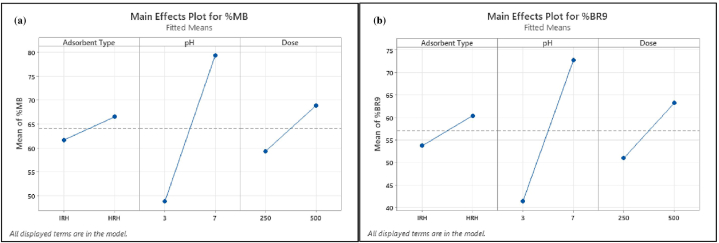
Main effect plots of dye removal for (a) MB (%) and (b) BR9 (%) during binary biosorption.

Main effect plots reveal which factors exhibit the greatest impact on the response (i.e., removal efficiency) (Fig. 14(a) and (b)). Each factor level affects response differently. The impact on the response is small when the slope is close to zero [77]. Thus, pH displayed the strongest positive influence on removal efficiency, followed by adsorbent dose.

**Fig. 15:**
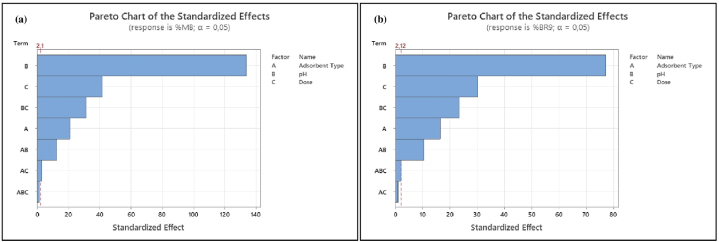
Pareto charts of dye removal for (a) MB (%) and (b) BR9 (%) during binary biosorption.

**Fig. 16:**
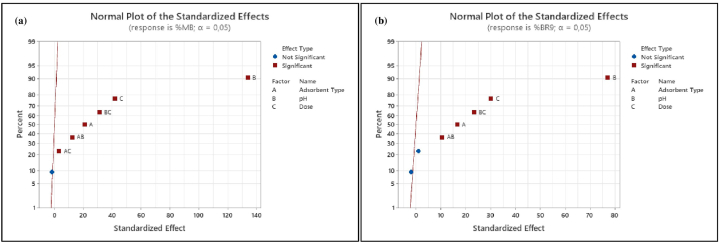
Normal plots of dye removal for (a) MB (%) and (b) BR9 (%) during binary biosorption.

Pareto charts (Fig. 15(a) and (b)) were explored to illustrate information obtained from normal plots (Fig. 16). Values to the right of the reference line (2.1 for %MB and 2.12 for %BR9) were significant. The vertical line indicates the minimum effect degree for a 95% confidence level [78]. Main factors (adsorbent type, pH, and dose) for both dyes and their interactions were significant. However, three-way interactions and adsorbent type*dose in BR9 (Fig. 15(b)) were insignificant.

We considered normal plots to assess each factor and interaction (Fig. 16(a) and (b)). Normal plots are employed to discriminate real effects from chance results. Each factor is defined as a point on the normal plots. A point located farthest from the line indicates the highest impact [75,77]. Normal plots could be divided into two areas:factors in the area above a standardized impact of 50% demonstrate positive effects and vice versa [73].

Main factors (adsorbent type, pH, and dose) display positive values and are far from the red line. Interactions between main factors show a similar result. However, interactions between adsorbent type*pH*dose for both dyes and adsorbent type*dose in BR9 present negative and insignificant impacts. Normal probability plots are consistent with Pareto charts (Fig. 15).

**Fig. 17:**
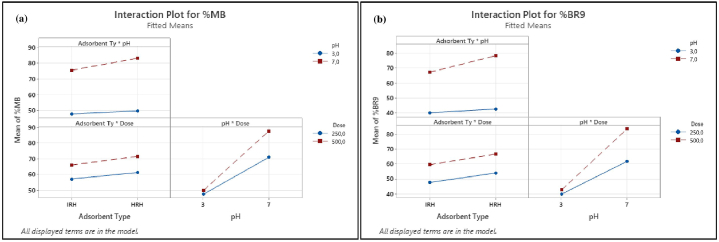
Interaction plots of dye removal for (a) MB (%) and (b) BR9 (%) during binary biosorption.

Interaction plots for pH*dose, adsorbent type*dose, and adsorbent type*pH interactions were also considered (Fig. 17(a) and (b)). When plot lines are not parallel, interactions between control factors are strong and vice versa [54]. Important interactions were observed between pH and dose for MB and BR9. Further, the interaction of adsorbent type and pH is less crucial, and the interaction between adsorbent type and dose can be characterized as minimal.

## 4 Conclusions

Removal of MB and BR9 in binary biosorption was investigated. Maximum removal percentages for MB and BR9 using HRH were 91.7% and 88.8%, respectively, and 83.8% and 78.2% using IRH, respectively. A BET multilayer isotherm best fit experimental data for binary biosorption. Binary biosorption of dyes followed the Elovich equation based on kinetic data. A factorial design analysis indicated that interactions among adsorbent type*pH*dose for both dyes were insignificant. Interactions between main factors (adsorbent type, pH, and dose) were significant for MB and BR9, except for two-way interactions (adsorbent type*dose) for BR9. HRH and IRH are inexpensive and environmentally friendly materials that can be successfully utilized as adsorbents for removing MB and BR9. These adsorbents should be further studied for dye removal from real/synthetic wastewater to evaluate practical applicability and considered the regeneration–reusability of adsorbent. In addition, to improve the application of adsorbent, the fixed bed column method can be utilized on a large scale for industrial purposes.

## Declarations

### Funding statement

The 10.13039/501100015763University of Szeged supported Open Access Funding (grant number: 6230).

## Author contribution statement

Hadid Sukmana: Conceived and designed the experiments; Performed the experiments; Analyzed and interpreted the data; Wrote the paper.

Gergő Ballai: Analyzed and interpreted the data; Contributed reagents, materials, analysis tools or data.

Tamás Gyulavári: Erzsébet Illés: Contributed reagents, materials, analysis tools or data; Wrote the paper.

Gábor Kozma: Zoltán Kónya: Contributed reagents, materials, analysis tools or data.

Cecilia Hodúr: Conceived and designed the experiments; Analyzed and interpreted the data; Contributed reagents, materials, analysis tools or data; Wrote the paper.

## Data availability statement

Data included in article/supp. material/referenced in article.

## Declaration of competing interest

The authors declare that they have no known competing financial interests or personal relationships that could have appeared to influence the work reported in this paper
